# Potential Application of Muscle Precursor Cells from Male Specific-Pathogen-Free (SPF) Chicken Embryos in In Vitro Agriculture

**DOI:** 10.3390/ani13111887

**Published:** 2023-06-05

**Authors:** Won-Seok Ju, Kangmin Seo, Bo-Ram Lee, Mi-Ryung Park, Min-Gook Lee, Sung-June Byun, Hyeon Yang, Youngim Kim, Sun-A Ock

**Affiliations:** Animal Biotechnology Division, National Institute of Animal Science (NIAS), Rural Development Administration (RDA), 1500 Kongjwipatjwi-ro, Wanju-gun 565-851, JB, Republic of Korea

**Keywords:** specific-pathogen-free (SPF) chicken embryo, cultured meat, myogenic precursor cells, sex, poultry, *Salmonella*

## Abstract

**Simple Summary:**

The present study examined the potential benefits of male specific-pathogen-free (SPF) White Leghorn embryos in cellular agriculture for sustainable and ethical poultry meat production—addressing traditional farming challenges, including disease outbreaks of *Salmonella* and *Avian* influenza. We generated myogenic precursor cells (MPCs) from the thigh muscles of embryos from SPF White Leghorns that tested negative for *Salmonella*. When male and female embryonic MPCs were cultured in vitro, it was confirmed that genetic sex did not affect the expression of factors related to myogenic regulation factors. Therefore, MPCs from male SPF-laying chicken embryos are promising for developing clean animal-cell-derived protein sources via resource recycling.

**Abstract:**

This study examined the potential benefits of male specific-pathogen-free (SPF) White Leghorn embryos in cellular agriculture for sustainable and ethical poultry meat production—addressing traditional farming challenges, including disease outbreaks of *Salmonella* and Avian influenza. We isolated myogenic precursor cells (MPCs) from the thigh muscles (*Musculus femoris*) of 12.5-day-old embryos from 10 SPF White Leghorns that tested negative for *Salmonella*. We randomly selected MPCs from three males and three females, isolated them using a modified pre-plating (pp) method, and compared their in vitro development. After 1 h (pp1) and 2 h (pp2) of incubation, they were transferred to a new dish to remove fast-adhering cells and cultured (pp3). Isolated MPCs had a 69% positive reaction to Pax7. During proliferation, no differences were observed in *PAX7, MYF5*, or *MYOD* expression between the male and female MPCs. However, after five days of differentiation, the expression of late myogenic factors—*MYOG* and *MYF6—*significantly increased in all MPCs. Notably, *MYOG* expression was 1.9 times higher in female than in male MPCs. This impacted *MYMK*’s expression pattern. Despite this, the myotube fusion index did not differ between the sexes. Muscle cells from male SPF-laying chicken embryos are promising for developing clean animal-cell-derived protein sources via resource recycling.

## 1. Introduction

The projected doubling of global meat demand by 2050 is raising significant concerns regarding the sustainability and availability of animal protein [1,2]. In addition, traditional farming systems with domestic animals raise concern because the energy required for production exceeds the energy obtained from intake [3]. The production of animal proteins through in vitro cell culture has attracted attention as an alternative to these challenges in domestic animal farming systems [4,5].

Cultured meat, also known as cultivated meat, is produced in vitro by isolating cells from tissues of domestic animals [6,7,8]. Among livestock, chickens have been used as in vitro or in vivo experimental models in various fields, including basic sciences, the food industry, and pharmaceutical fields for human beings [9]. Despite its importance, *Salmonella*, a pathogen in the digestive tract or feces of chickens, often causes food-borne human diseases and adversely affects the economy due to reduced chicken productivity [10]. Therefore, in vitro cell culture engineering studies using embryos or chickens produced under specific-pathogen-free (SPF) conditions are required to overcome these limitations.

Most livestock possess XX sex chromosomes or XY (male heterogamety), whereas chickens possess ZW (female heterogamety) or ZZ [11]. The genetic factors related to functional differences in proliferation and differentiation determined by sex in chickens are not yet clearly understood. Generally, genotype and sex are essential for chicken productivity, and males are more productive than females in several chicken breeds [12]. Sex hormones, such as androgens, secreted by genetically determined sex, positively affect the proliferation and differentiation of skeletal muscle satellite cells with androgen receptors in livestock and humans in vivo [13,14]. However, the effects of androgens on the in vitro culture of satellite cells/myoblasts are controversial [15].

We aimed to develop a safe source of animal-cell-cultured meat using discarded chicks and embryos from laying hens with productivity problems, while ensuring it does not pose a risk of severe food poisoning in humans. To achieve this, we characterized skeletal-muscle-derived myogenic progenitor cells (MPCs) from SPF chicken embryos. Additionally, we investigated how sex influences muscle cell growth and differentiation by comparing the expression of genes encoding myogenic regulatory factors (MRFs) in cultured male and female MPCs.

## 2. Materials and Methods

### 2.1. Ethics Statements

All experiments and instructions in this study were performed in accordance with the guidelines for the use of animals in research as stated by the Institutional Animal Care and Use Committee (IACUC) of the National Institute of Animal Science (Approval number NIAS20222452) in the Rural Development Administration (RDA), Republic of Korea.

### 2.2. Reagents and Media

Unless otherwise specified, all reagents and chemicals were purchased from Sigma-Aldrich (St. Louis, MO, USA), and the study medium was obtained from Invitrogen-Gibco (MD, USA).

### 2.3. Preparation of Experimental Embryos

The 10 embryos used in this study were obtained from SPF White Leghorn lines (Namdeok SPF, Seoul, Republic of Korea). The collected eggs were steadily maintained at an egg-turning frequency of 48 times per day (30 min/1 time) at 37 °C under a relative humidity of 55–60% in an incubator chamber until further use.

### 2.4. Microbial Testing Using the SS MC-Media Pad

Embryos from SPF White Leghorn and backyard hens were assessed for microbial contamination using a commercially available SS MC-Media Pad (JNC Corporation, Tokyo, Japan) following the manufacturer’s instructions. The egg surface and egg white and yolk mixture were tested and incubated for 48 h after inoculation. The light blue colonies indicated *Salmonella*, whereas the purple colonies indicated *Escherichia coli* (*E. coli*).

### 2.5. Isolation and Culture of Myogenic Precursor Cells (MPCs)

**Figure 1:**
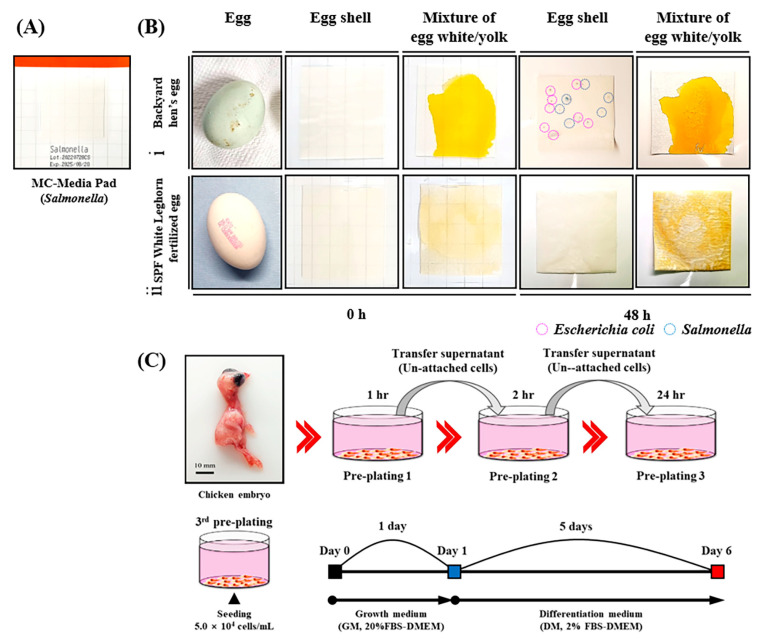
Schematic overview of MPCs’ isolation from SPF chicken embryos. A microbial contamination test was performed using the SS MC-Media Pad (**A**) in the backyard hen’s eggs (**Bⅰ**) and the specific-pathogen-free (SPF) White Leghorn embryos (**Bⅱ**). The egg surface and egg white and yolk mixture were tested and incubated for 48 h after inoculation. The light-blue colonies (blue circles) indicate *Salmonella*, whereas the purple colonies (light purple circles) indicate *Escherichia coli* (*E. coli.*). (**C**) Myogenic precursor cells (MPCs) were isolated from the thigh muscles (*Musculus femoris*) of chicken embryos at E12.5 using a pre-plating protocol. The supernatant containing dissociated cells was transferred to a new dish. At passage 3, cells were seeded at a 5.0 × 10^4^ cells/mL concentration in 24-well culture plates and cultured in a differentiation medium (DM; 2% FBS-ADMEM supplemented with 1% PS) for five days after one day of proliferation before being used in the experiments.

The eggshells were cleaned using an alcohol swab to collect SPF White Leghorn embryos. MPCs were surgically excised from the lower extremities of the chicken embryos (embryonic day ~12.5 (E12.5)) and then thoroughly decontaminated thrice with Dulbecco’s phosphate-buffered saline without calcium and magnesium (DPBS; Invitrogen-Gibco, Gibco, Grand Island, NY, USA) containing 1 × Antibiotic-Antimycotic (Invitrogen-Gibco, Grand Island, NY, USA) and 1× Penicillin-Streptomycin (PS; Invitrogen-Gibco, Grand Island, NY, USA). Furthermore, 200 mg of skeletal muscle tissues from both thighs of the chicken embryos was finely cut, 2 mL of 0.25% collagenase type Ⅰ (STEMCELL technology, Vancouver, BC, Canada) was consecutively added, then the tissues were maintained at 37 °C under 5% CO_2_ in a humidified incubator chamber until further experiments. After the previous disassociation, the tissues underwent 10 repeated passages through 10 mL of an 18-gauge needle to mechanically dissociate the cell aggregates. The tissue fragments were then filtered through 40 and 100 µm cell strainers (Nylon mesh, Somerville, BD Falcon, Corning, Big Flats, NY, USA) to recover the fraction. MPCs were isolated from the separated cell suspensions via a pre-plating protocol [16] using the difference in cell attachment speed, as indicated in Figure 1. After, suspended cells were ultimately plated in a new T-flask coated with collagen (75 cm^2^, Corning, Grand Island, NY, USA) and maintained in an F-10 medium (Invitrogen-Gibco, Grand Island, NY, USA) supplemented with 20% fetal bovine serum (FBS; heat-inactivated, Invitrogen-Gibco, Grand Island, NY, USA), 1% PS, and 2.5 ng/mL of basic fibroblast growth factor (bFGF; Invitrogen-Gibco, Grand Island, NY, USA) at 37 °C under 5% CO_2_ in a humidified incubator chamber. After 1 h of incubation, the supernatant containing the dissociated cells (pre-plate 1, pp1) was transferred to a new T_75_-flask coated with collagen and cultured. After 2 h, the supernatant containing dissociated cells (pre-plate 2, pp2) was transferred to a new T_75_-flask coated with collagen and cultured (pre-plate 3, pp3).

### 2.6. Cell Culture

MPCs (5 × 10^4^ cells/mL) in pp3 were seeded and maintained in a 24-well cell culture plate (Corning, Big Flats, NY, USA) coated with collagen type Ⅰ (Cellmatrix Type Ⅰ-A, Nitta Gelatin Inc., Osaka, Japan) with the growth medium (GM; 20% FBS-advanced Dulbecco’s modified eagle’s medium (ADMEM; Invitrogen-Gibco, Grand Island, NY, USA) supplemented with 1 × PS). After 24 h, the cultured MPCs were replaced with the DM (2% FBS-ADMEM with 1 × PS), maintained for five days, and then replaced with fresh DM every two days.

Chicken fibroblast DF-1, mouse myoblast—C2C12, and quail myoblast—QM7 cells were obtained from the American Type Culture Collection (ATCC; Manassas, VA, USA). Cells were maintained in ADMEM supplemented with 10% FBS, 1 × GlutaMAX (Invitrogen-Gibco, Grand Island, NY, USA) and 1 × PS (Invitrogen Gibco, Grand Island, NY, USA) at 37 °C under 5% CO_2_ in a humidified incubator chamber until further use.

### 2.7. Sex Determination

The sexes of the chicken embryos were determined using the method described by He et al. [17]. Genomic DNA was extracted and amplified from the excess tissue obtained after harvesting the leg muscles of chicken embryos using a DNeasy Blood & Tissue Kit (Qiagen GmbH, Hilden, NRW, Germany). Thereafter, 1 µL of nucleic acid (100 ng) and known primers [17] were used for polymerase chain reaction (PCR) under the following conditions: for *SWIM* gene encoding zinc finger SWIM domain-containing protein 6-like present on W-chromosome and *12S* gene, denatured at 94 °C for 2 min, followed by 30 cycles of annealing and extension at 94 °C for 30 s, 55 °C for 30 s, 72 °C for 30 s, and 72 °C for 5 min. The PCR products (*SWIM*: 212 bp and *12S*: 131 bp) stained with ethidium bromide were subjected to electrophoresis on 2% agarose gel using a UV transilluminator.

### 2.8. Myotube Fusion Index (MFI)

MPCs were fixed in 10% formalin (Sigma-Aldrich, St. Louis, MO, USA) for 30 min at room temperature and rinsed thrice in DPBS after being kept clean for five days in a humidified incubator chamber. Cells were permeabilized by washing in DPBS containing 0.5% Triton X-100 (Bio-Rad Laboratories Inc., Hercules, CA, USA) for 10 min and then rinsed in DPBS thrice. Consequently, MPCs were blocked with DPBS containing 1% bovine serum albumin (BSA; Sigma-Aldrich, St. Louis, MO, USA), 3% goat normal serum (Abcam Inc., Cambridge, UK), and 0.5% Tween 20 (Sigma-Aldrich, St. Louis, MO, USA) at room temperature for 1 h. Myosin-heavy-chain-positive cells containing three or more nuclei were identified as myotubes. The ratio of myotube fusion to the number of myotubes was further investigated. Primary antibody monoclonal anti-myosin (1:400) (Sigma-Aldrich, St. Louis, MO, USA) was added and incubated at 4 °C overnight. The samples were then incubated with Alexa Fluor secondary antibody goat anti-mouse IgM (1:400) (Invitrogen, Waltham, MA, USA) for 1 h in the dark (Table 1). For nuclear counterstaining, 1 µg/mL of 4′,6-diamidino-2-phenylindole (DAPI) was used for 15 min. Stained samples were rinsed twice with DPBS and visualized under a fluorescence reverse microscope (Leica DMI 6000 B, Leica, Wetzlar, Germany). For myotube fusion index (MFI), myosin-heavy-chain-positive cells with three or more nuclei were counted in myotubes from 10 images per well, and this was used to calculate the number of nuclei in the myotube/total number of nuclei × 100 (%), utilized as an indicator of MPC differentiation [7,18]. Two co-workers independently counted.

### 2.9. Confirmation of Skeletal Muscle Stem Cell Ability

The protein expression of the transcription factors of muscle satellite cells/myoblasts, Pax7, and Myod were analyzed in MPCs, with myoblast cells (C2C12 and QM7) and DF-1 as the positive and negative control, respectively. Each sample was cultured on collagen-coated cover glass in a 4-well dish for three days and fixed with 3.7% formalin. They were blocked in DPBS containing 3% BSA and 0.3% Triton X-100 for 1 h at room temperature. The cells were rinsed twice with DPBS every 5 min. Detailed information regarding the antibodies used in this study is presented in Table 1. For nuclei staining, cells were stained with 1 µg/mL of DAPI solution for 30 min at room temperature in the dark and then rinsed twice with DPBS containing 1% BSA. They were mounted using Vectashield solution (Vector Laboratories, Inc., Burlingame, CA, USA). Finally, cells were observed under a fluorescence microscope (Leica DMI 6000 B; Leica, Wetzlar, Germany) in the dark. Pax7 expression was quantified by dividing the number of FITC-positive nuclei by the total number of nuclei and converting it into a percentage. In this experiment, three regions were randomly observed per sample, with five replicates performed. Data are presented as mean ± standard error of the mean (SEM).

### 2.10. Real-Time-Quantitative PCR (RT-qPCR)

Total RNA was isolated using the RNeasy Mini kit (Qiagen GmbH, HD, Germany) before and after MPC differentiation for five more days. cDNA was synthesized from the extracted total RNA (500 ng) using Omniscript Reverse Transcriptase (Qiagen GmbH, Hilden, NRW, Germany) with anchored Oligo dT 20 (Invitrogen, Waltham, MA, USA) at 37 °C for 60 min according to the manufacturer’s instructions. Specific primers were designed using Primer Express 3.01 software (Applied Biosystems, Foster City, CA, USA); detailed primer information is presented in Table 2. RT-qPCR was performed with the SYBR Green PCR Master Mix (Applied Biosystems, CA, USA) using a StepOnePlus Real-Time PCR system (ABI, NY, USA). The TATA-Box Binding Protein gene (*TBP*) was the internal control. Relative quantification (RQ) of the gene expression was calculated using the delta-delta Ct method, and the data are presented as RQ. All quantification was normalized to the housekeeping gene *TBP*. Biological replicates were collected from three independent chicken embryos of each sex.

### 2.11. Statistical Analysis

All data were analyzed for statistical significance using one-way ANOVA, post hoc Tukey’s multiple range test, or LSD test using the Statistical Package for the Social Sciences statistics software (IBM SPSS Corp., Armonk, NY, USA) (ver. 25). A *p*-value of <0.05 was significant in this study. All experiments were performed in five replicates.

## 3. Results

### 3.1. Microbial Contamination Testing

In this study, SPF White Leghorn and backyard hen embryos were tested for pathogens, and *Salmonella* (light blue) and *E. coli* (purple) colonies were detected on the eggshells of some backyard hen eggs. In contrast, no colonies of either pathogen were found in the eggshells, egg white, or yolk of SPF White Leghorn embryos (Figure 1A).

### 3.2. Morphology and Sex Determination of Chicken Embryos

**Figure 2:**
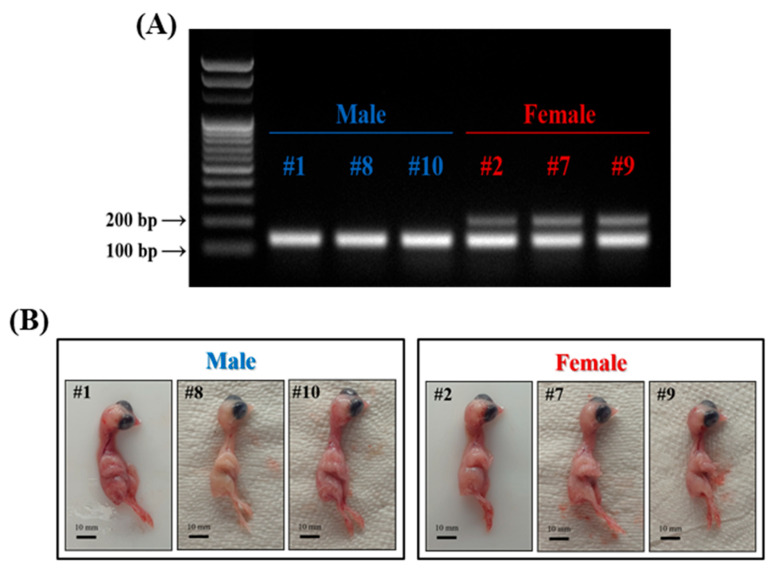
Sex determination of chicken embryos. (**A**) Reverse-transcription PCR analysis for gene amplification related to sex was performed. In females, two bands are expressed: one corresponding to the SWIM gene (212 bp) located on the W-chromosome, and another band at 12S (131bp), which serves as a reference gene for chickens. On the other hand, males only exhibit a single band for 12S. (**B**) Ten chicken embryos were analyzed using PCR to determine their sex, and three male and three female embryos were selected for further study. The selected embryos are presented in the image with embryo identification numbers indicated with #. The scale bars indicate 10 µm.

Anatomically, chicken embryos aged 10–13 days were confirmed to be hairless and had significantly more developed thigh (*Musculus femoris*) than breast (*Pectoralis major*) muscles, as illustrated in Figure 1 and Figure 2. Therefore, the thigh muscle is the most suitable cell source for supplying skeletal-muscle-derived MPCs. Thigh muscles were harvested from ten 12.5-day-old chicken embryos to isolate MPCs. The surplus tissues were used to identify the embryos’ sex.

*SWIM*, specifically on the W chromosome of female chickens (ZW), was determined using a genomic polymerase chain reaction. *SWIM* (212 bp) increased in chicken embryos presumed to be females (Figure 2A). Contrastingly, its expression did not increase in chicken embryos presumed to be males. Based on these results, three male (embryos #1, #8, and #10) and female (embryos #2, #7, and #9) chicken embryos (E12.5) were selected randomly and used for isolating and analyzing MPCs (Figure 2B). No differences in size were observed between male and female embryos.

### 3.3. Characterization of MPCs

**Figure 3:**
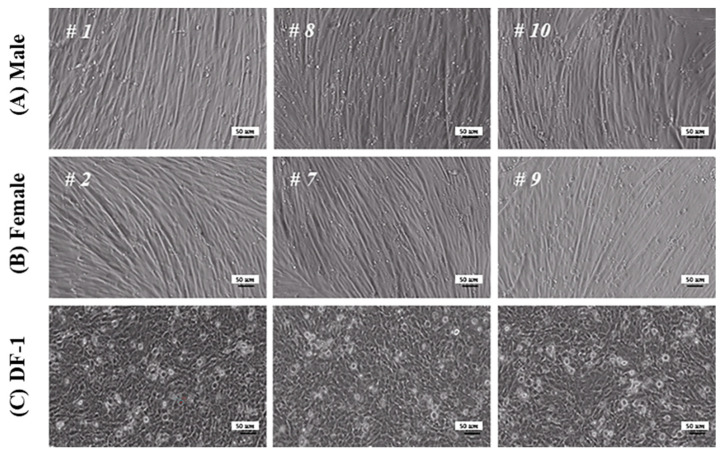
Morphological changes in in vitro culture of male and female MPCs derived from SPF chicken embryos. MPCs isolated from male (**A**) and female (**B**) chickens were cultured in the GM for one day and in DM for five days. Chicken fibroblasts (DF-1) (**C**) used as a negative control were cultured under the same conditions. The “#” symbol was used to generate a unique identification number for the embryos. Each scale bar indicates 50 µm.

The MPCs isolated from the randomly selected chicken embryos were successfully cultured and differentiated (Figure 3). They were well developed as long spindle-shaped myotubes, compared to the negative control group (DF-1), regardless of sex. In addition, no differences were observed among the embryos.

### 3.4. Confirmation of Skeletal Muscle Stem Cell Factors—PAX7 and MYOD

**Figure 4:**
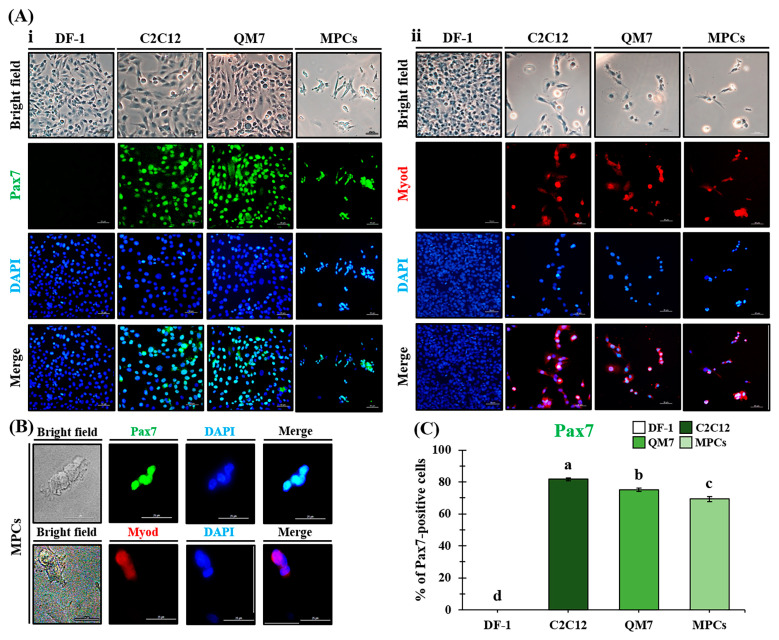
Expression of satellite cell/myoblast transcription factors in MPCs derived from SPF chicken embryos. Expression of Pax7 (**Aⅰ**) and Myod (**Aⅱ**) in various cells, with QM7 and C2C12 as positive controls, DF-1 as a negative control, and MPCs. Scale bar: 50 μm. (**B**) Expression of Pax7 or Myod in the nuclei of MPCs. They were stained using PAX7 mouse and Myod monoclonal antibodies as primary antibodies and goat anti-mouse IgG FITC and IgG (H+L) Alexa Fluor Plus 555 as secondary antibodies. The green fluorescence represents Pax7 staining, while the red fluorescence indicates Myod staining. Nuclei were stained with DAPI (blue). Scale bar: 25 μm. (**C**) Comparison of the number of Pax7-positive cells in various cells. Two independent co-workers counted. Different letters denote significant differences within each group (assessed using one-way ANOVA followed by the Tukey-HSD multiple range tests, SPSS 25, ^a–d^ *p* < 0.05). Each bar indicates the fibroblast DF-1 (white), mouse myoblasts C2C12 (dark green), quail myoblasts (green), and chicken MPCs (light green).

Pax7 and Myod expression in MPC was analyzed by comparing the positive controls C2C12 and QM7 with the negative control chicken fibroblast, DF-1, as illustrated in Figure 4. Positive reactions for Pax7 and Myod were confirmed in three cell types, C2C12, QM7, and MPCs, despite the different degrees of expression. Regarding transcription factors of the muscle satellite cells Pax7 and Myod, except for the negative control DF-1, PAX7 was strongly expressed in the nucleus in all groups. However, Myod was weakly expressed in the nucleus. MPCs, when compared to Pax7-positive C2C12 and QM7 cells, exhibited a small and round cell size of approximately 10 μm (Figure 4B). The quantification of PAX7 expression across all groups revealed percentages of 81.69 ± 0.78, 75.03 ± 1.08, and 69.31 ± 1.63 for C2C12, QM7, and MPC, respectively (Figure 4C). Based on the above results, we confirmed that chicken MPCs were successfully recovered from chicken embryos, and the following experiments were conducted.

### 3.5. Genes Involved in the Growth and Differentiation of MPCs

**Figure 5:**
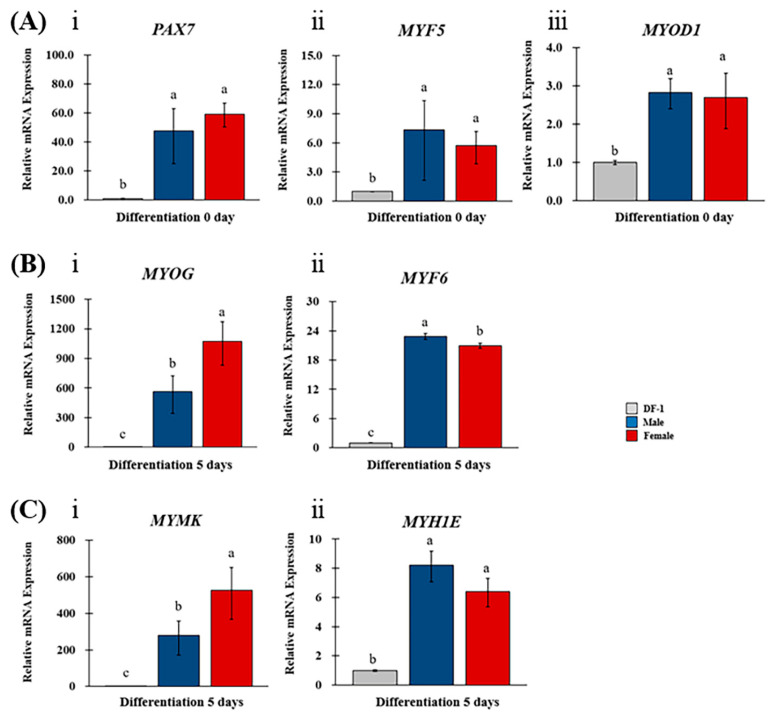
Comparing the expression of myogenesis-related markers using the sex of MPCs derived from SPF chicken embryos. The expression of myogenesis-related factors was analyzed in MPCs cultured in vitro using real-time PCR. (**A**) *PAX7* (**ⅰ**), *MYF5* (**ⅱ**), and *MYOD1* (**ⅲ**) levels were examined in cells cultured for one day in a GM. *MYOG, MYF6, MYMK*, and *MYH1E* levels were analyzed in cells cultured in GM for one day, followed by an additional five days in DM. The expression of late myogenic regulators (*MYOG* (**ⅰ**) and *MYF6 (***ⅱ**)) and myotube-formation-related factors (*MYMK* (**ⅰ**) and *MYH1E* (**ⅱ**)) were also examined—(**B**) and (**C**), respectively. Error bars represent the RQ of minimum and maximum expression levels around the mean RQ expression. Different letters denote significant differences within each group (assessed using the one-way ANOVA followed by the Tukey-HSD multiple range test, SPSS 25, ^a–c^ *p* < 0.05). All experiments were performed in triplicate. The blue and red bars indicate male and female MPCs, respectively.

We analyzed the expression of the related genes *PAX7*, *MYF5*, and *MYOD* in isolated MPCs to confirm the potential proliferative capacity of satellite cells and myoblasts and to determine whether they exhibit the characteristics of MPCs (Figure 5A). Regardless of sex, *PAX7* was upregulated by over 47.5-fold in MPCs compared to in DF-1 cells. *MYF5* and *MYOD* were upregulated in all MPCs compared to DF-1 cells; however, not to the same extent as *PAX7*. No significant differences existed in the expression of these three genes between the sexes (*p* < 0.05).

We analyzed the expression of the major myogenic regulatory factors *MYOG* and *MYF6* in differentiated MPCs (Figure 5B). Compared with DF-1, *MYOG* was dramatically upregulated in male and female MPCs, with 565.6-fold and 1074.3-fold increases, respectively. Additionally, the expression of *MYOG* was 1.9 times higher in female than in male MPCs. Similarly, *MYF6* was increased in all MPCs than in DF-1 cells. However, males had a 1.1 times higher increase than females.

Finally, *MYMK* and *MYH1E,* involved in myotube formation, were analyzed in the MPCs (Figure 5C). Comparing *MYMK* expression between MPCs and DF-1 cells, it was upregulated by 280.26-fold in male MPCs and 526.96-fold in female MPCs, similar to the *MYOG* expression pattern, with a 1.9-fold increase in females compared to that in males. Compared to the *MYH1E* expression in DF-1 cells, *MYH1E* was also upregulated in all MPCs and did not differ significantly between sexes.

### 3.6. Effect of Sex on Myotube Fusion Index (MFI) of MPCs

**Figure 6:**
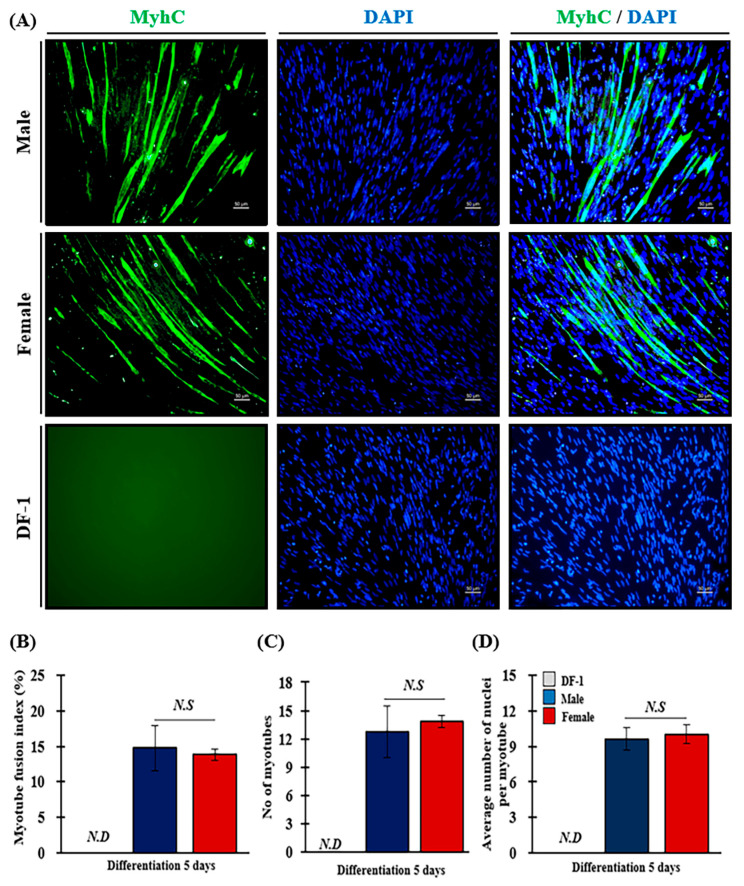
Comparing MFI of using the sex of MPCs derived from SPF chicken embryos. (**A**) MPCs were differentiated in vitro for five days, and myotube formation was analyzed using immunofluorescence staining for Myhc. DF-1 was the negative control. Immunofluorescence staining for Myhc was performed using monoclonal anti-myosin and goat anti-mouse IgM Alexa Fluor™488 as the primary and secondary antibodies, respectively. Nuclei were stained with DAPI (blue). The scale bars indicate 50 µm. The results from calculating the MFI are indicated as follows: MFI (%) (**B**), number of myotubes (**C**), and average number of nuclei per myotube (**D**). The experiments were performed in triplicate. The blue and red bars indicate male and female MPCs, respectively. The results were presented as the mean ± standard error (SEM). Student’s *t*-tests were performed to compare differences between groups (*p* < 0.05). *N.D* and *N.S* represent non-detectable and non-significant differences (*p* > 0.05), respectively (*p* > 0.05).

Many elongated, cylindrical, and multinucleated myotubes (green) were observed in male and female MPC (Figure 6A). DF-1 cells did not exhibit a positive green signal. No difference was observed in the myotube formation competence of MPCs by sex.

For precise analysis, differentiated MPCs were quantified using MFI analysis (Figure 6B and Table 3). MFI did not significantly differ between male (14.75 ± 3.15%) and female MPCs (13.82 ± 0.85%). No significant differences were observed between the sexes regarding the total number of nuclei in the myotubes that affected the MFI index due to individual differences (Table 3). The number of confirmed myotubes was 12.75 in male MPCs sand 13.82 in female MPCs, respectively. The average nuclei number per myotube in male and female MPCs was 9.61 ± 0.95 and 10.03 ± 0.79, respectively.

## 4. Discussion

Livestock is a major source of protein for humans. Chickens and eggs are the major sources of protein worldwide. However, they can sometimes cause fatal diseases through viral and bacterial infections. *Salmonella*, transmitted through contact with chicken feces, harms human public health and food safety, and avian influenza reduces chicken populations [10,19,20]. We aimed to develop a clean alternative protein supply method using SPF embryos from laying hens. Additionally, we aimed to utilize discarded resources, such as male chicks, to develop alternative protein sources.

*Salmonella Enteritidis* is a major pathogen responsible for human food poisoning and reduces poultry farming productivity. Notably, this pathogen can be detected in various egg parts, including egg yolks, egg whites, and eggshells, possibly contaminating eggs produced by infected hens [10]. SPF chicken-embryo-derived skeletal muscle tissue, as a cell source for in vitro clean cellular agriculture, is a novel approach made possible by its previous use in disease, pathogen infection, vaccine development, and new drug development models for humans [21]. By extending its use to in vitro clean cell farming, we could create a more sustainable and ethical source of meat for human consumption while reducing the negative impacts of traditional livestock farming on the environment and animal welfare.

In vitro and in vivo studies comparing the growth and differentiation abilities of muscle stem cells from embryos and adults have yielded conflicting results regarding their age-related regenerative potential [22]. Embryonic muscle tissue contains more muscle stem cells, such as myosatellite cells and myoblasts, than adult muscle tissue [23,24,25]. PAX7, a marker of satellite cells, muscle stem cells, and myoblasts, promotes myocyte proliferation via asymmetric division to generate PAX7+/MYOD+ cells. However, its expression is influenced by factors such as chicken breed, age, and embryonic age and declines with aging [23,24,25]. Although not of the same species, our previous study revealed that the expression of Pax7 in 2-year-old Korean Bulls was relatively low compared to another group using a 15-day-old Simmental calf [8,26]. Our study focused on isolating fetal myoblasts, typically found at 8–12 days of age [27,28]. Our study successfully isolated fetal satellite cells/myoblasts expressing PAX7, a marker of skeletal muscle progenitor cells, with a recovery rate of 69% and high purity, comparable to the positive control—C2C12/QM7. These cells can serve as valuable resources for cultured meat production. Furthermore, the real-time PCR results for *PAX7* and *MYOD* were consistent with the immunostaining data.

Furthermore, the growth rate difference between male and female broilers becomes more pronounced after the early growth stage [29,30,31,32]. Differences in muscle development patterns and organ weight emerge as early as the embryonic period. Despite these findings, a paucity of research currently exists on the in vitro proliferation and differentiation of sex-dependent MPCs derived from chicken embryos. Male embryos and fetuses, typically discarded by the laying hen industry, are of particular interest and potentially valuable sources of precursor cells for cultured meat production [17]. Our study uncovered a significant upregulation of late myogenic regulatory factors involved in muscle development in differentiated muscle progenitor cells in males and females. Particularly, females had approximately 1.9-fold higher *MYOG* expression than males, which was corroborated by the upregulation of *MYMK* in females. However, no significant differences were observed in *MYH1E* expression or the myotube fusion index (MFI) between the sexes, despite *MYOG* and *MYMK* upregulation in females. This may be attributed to individual variability in our study population. Further studies with larger sample sizes and more diverse populations are necessary to validate our findings.

Previous studies have revealed that MYOG expression is higher in female than that in male mice at 7 days of age; however, the opposite is observed in mice aged ≥40 days. This difference may be due to androgenic receptors on satellite cells; however, sex hormone production increases with sexual maturation, typically occurring between 30 and 60 days in mice. Thus, the effect of sex hormones on satellite cell activity may not be fully apparent at the ages we studied [19,33].

## 5. Conclusions

We investigated the potential use of SPF embryos of laying hens as a cell source for cultured meat production. We observed that utilizing discarded surplus resources could provide a sustainable and environmentally friendly alternative to traditional animal protein sources. Through MRFs and MFI expression, we established that male embryonic MPCs not exposed to male hormones had similar in vitro developmental abilities to female embryonic MPCs. Thus, SPF male embryos are expected to be a novel source of animal protein. Finally, we did not investigate the antibiotic capacity of the culture medium. However, we expect that this clean cell source will reduce the need for antibiotics in long-term cultures.

## Figures and Tables

**Table 1:** Antibodies used for the myotube fusion index and immunocytochemistry staining in this study.

Antibodies			Dilution Factor	Time
Name	Information			
Myhc	Primary	Monoclonal anti-myosin (Skeletal, Fast) IgG_1_ (Sigma-Aldrich, St. Louis, MO, USA)	1:400	Overnight
	Secondary	Goat anti-mouse IgM (Heavy chain) Cross-Absorbed Alexa Fluor™488 (Invitrogen, Waltham, MA, USA)	1:400	1 h
Pax7	Primary	PAX7 (Rhabdomyosarcoma Marker) Mouse Monoclonal Antibody [Clone PAX7/497] (NeoBiotechnology, Union city, CA, USA)	1:1000	Overnight
	Secondary	Goat anti-mouse IgG H&L (FITC) (Abcam Inc., Cambridge, UK)	1:1000	1 h
Myod	Primary	MYOD Monoclonal Antibody (5.8A) IgG1_κ_ (Invitrogen, Waltham, MA, USA)	1:1000	Overnight
	Secondary	Goat anti-mouse IgG (H+L) Alexa Fluor Plus 555 (Invitrogen, Waltham, MA, USA)	1:1000	1 h

Note: Myhc, myosin heavy chain; Pax7, paired box 7; Myod, myoblast determination protein; FITC, fluorescein isothiocyanate; IgG, immunoglobulin G; IgM, immunoglobulin M.

**Table 2:** Primers used for real-time quantitative PCR analysis.

Gene	Primer Sequence (5′→3′)	Accession Number	Product Size (bp)
*PAX7* (Paired box 7)	F: ATT AGC CGT GTG CTA CGC AT R: AGC CTT CAT CCA GCC TGT TC	NM_205065.1	142
*MYF5* (Myogenic factor 5)	F: GAG GAA CGC CAT CAG GTA CAT C R: AGT TCT CCA CCT GTT CCC TCA A	NM_001030363.1	62
*MYOD1* (Myoblast determination protein 1)	F: GCC GCC GAT GAC TTC TAT GA R: CAG GTC CTC GAA GAA GTG CAT	NM_204214.2	66
*MYOG* (Myogenin)	F: CGT GTG CCA CAG CCA ATG R: CCG CCG GAG AGA GAC CTT	NM_204184.1	63
*MYF6* (Myogenic factor 6)	F: GCG CCA TCA GCT ACA TCG A R: GCA TTT TGT CCT GCT GAT CCA	NM_001030746.1	66
*MYMK* (Myomaker)	F: TGC GCT ATG ACA TCC TGG AGT A R: GGG ACA CCC AGA TGG ACA GA	NM_001318457.1	63
*MYH1E* (Myosin heavy chain 1E)	F: TGG CAC AGT GGA CTA CAA CAT CT R: ACC ATA GGT GGC AAA CAG TAA GG	NM_001013397.2	127
*TBP* (TATA-box binding protein)	F: TTG TGT CCA CGG TGA ATC TTG R: TCG GGC ACG AAG TGC AAT	NM_205103.1	62

**Table 3:** Myotube fusion index (MFI) of male and female MPCs derived from SPF chicken embryos.

Embryo Description		No. of Total Nuclei	No. of Nuclei in Myotubes	MFI (%)	No. of Myotubes	Ave. of Nuclei in Myotubes
Male	#1	648.13	59.00	9.38	7.63	9.41
	#8	867.63	178.50	20.29	17.00	11.36
	#10	714.25	105.50	14.57	13.63	8.07
Mean		743.33	114.33	14.75	12.75	9.61
SEM		65.01	34.78	3.15	1.84	0.95
Female	#2	983.50	119.63	12.16	13.13	9.43
	#7	842.13	138.63	14.33	15.13	9.07
	#9	815.50	134.63	14.98	13.25	11.60
Mean		847.04	130.96	13.82	13.83	10.03
SEM		19.78	5.78	0.85	0.65	0.79

Note: MFIs were calculated as the number of nuclei in myotubes/total number of nuclei × 100 (%). Two co-workers independently counted. Abbreviations: Ave, average; No, number; SEM, standard error of the mean. The “#” symbol was used to generate a unique identification number for the embryos.

## Data Availability

All data generated or analyzed during this study are included in this published article.
